# Quantifying population level hypertension care cascades in India: a cross-sectional analysis of risk factors and disease linkages

**DOI:** 10.1186/s12877-022-02760-x

**Published:** 2022-02-04

**Authors:** Ajinkya Kothavale, Parul Puri, Purvi G. Sangani

**Affiliations:** International Institute for Population Sciences, Govandi Station Road, Deonar, Maharashtra 400088 Mumbai, India

**Keywords:** Elderly, Hypertension, India, LASI, Uncontrolled blood pressure

## Abstract

**Background:**

Hypertension is associated with higher morbidity and mortality burden, and is reported to pose severe repercussions on those above the age of 60 years. Despite the growing concern, empirical evidence providing nationally representative estimates of hypertension care cascades for the elderly population are inadequate in India. Therefore, the present study aims to quantify the magnitude of hypertension care cascades, identify the co-morbidities attributed to hypertension and recognize lifestyle modifications to reduce the instances of uncontrolled blood pressure among the elderly population in India.

**Method:**

This study employed data on 28,109 elderly individuals from the Longitudinal Ageing Study in India, 2017-18. Descriptive and multivariable analyses were performed to identify the burden and correlates of hypertension and uncontrolled blood pressure levels. Population Attributable Risk was computed to identify deteriorating health implications and recognize viable solutions to improve the situation.

**Results:**

The findings suggest that elderly experiences loss at all stages of hypertension care, namely, at the level of measured hypertension (72.5%), diagnosis/awareness (57.3%), treatment (50.5%), and control (27.5%). The highest dip was observed at the level of blood pressure control. The findings hint towards the linkages between socio-economic, demographic, and lifestyle factors with hypertension and uncontrolled blood pressure levels. Caste, religion, living arrangement, MPCE quintile, residence, family history of hypertension, working status, and alcohol consumption were the significant predictors of uncontrolled hypertension. The findings quantified the proportion of diseased cases attributed to hypertension, and highlighted essential contributors of overall and uncontrolled hypertension.

**Conclusions:**

There is an urgent need to improve access to cost-effective anti-hypertensive prescriptions to curtail the increasing burden of uncontrolled blood pressure and some other co-morbid diseases. Thus, if apprehended cautiously, findings from this study can serve to design practical approaches aimed at control, prevention, and management of hypertension among the elderly population of India.

**Supplementary Information:**

The online version contains supplementary material available at 10.1186/s12877-022-02760-x.

## Background

Rapid demographic and epidemiological transitions, high economic growth, and globalization have resulted in lifestyle modifications globally. These include changes in food habits, low levels of physical activity, consumption of alcohol, tobacco, and increasing body mass index (BMI) across the globe [1]. This transition can be hypothesized as a significant precursor to the burden of taxing Non-Communicable Diseases (NCDs) and hypertension among the adult and geriatric population [1]. As per the recent estimates, 1.13 billion people are affected with hypertension [2], with a prevalence of 22.1% globally [2]. The global projections have estimated that hypertension will affect 1.56 billion individuals by 2025 [3].

Existing literature has proposed a statistically significant association of hypertension with heart, brain, and kidney diseases. Hypertension is an important risk factor for cardiovascular morbidity and mortality, contributing around 25% of the NCDs burden globally [2]. A significant proportion of cardio-renal diseases like atrial fibrillation, chronic kidney disease, and non-cardio-vascular diseases such as oral health disorders, dementia, cancer, and reduced bone density can also be attributed to elevated blood pressure (BP) levels [4]. Due to the wide range of deteriorating health outcomes, raised BP affects more than two-thirds of individuals aged 65 years and above. Thus, hypertension is one of the significant public health concerns globally [1, 5].

Thus, Sustainable Development Goals (SDGs) recommended the reduction of hypertension prevalence by 25% by 2025 [2]. The World Health Organization suggested timely detection, effective screening, treatment, and palliative care, as critical strategies to combat accelerating NCD burden [6]. Patients unaware of their elevated BP levels develop life-threatening complications [7], and hypertension is termed as ‘silent killer’ [8]. Ongoing research has shown an evident decline in the prevalence rates of uncontrolled hypertension in developed countries over the past four decades [9]. However, there has been a significant increase in the burden of uncontrolled hypertension in low-and-middle-income countries (LMICs), hailing from Africa, South Asia, central and eastern Europe [9]. In addition, BP control rates are meager (<30%) among individuals who are diagnosed and treated for hypertension [9]. Existing literature estimates that raised blood pressure accounts for 57% of all stroke deaths and 24% of all coronary diseases in India [10]. In a population-based study on the urban geriatric population of South India, it was found that hypertension was controlled in about 46% of individuals who were taking treatment, amongst the 80% having been diagnosed with hypertension [11]. Another study conducted in India reported around 6% of the working-age men were controlled for elevated BP levels, afflicting an unmet need for hypertension care of around 94% [12].

India holds a disproportionately high burden of mortality and morbidity associated with hypertension, increasing over time. These deteriorating implications are far worse for individuals over 60 years [13]. A recently published study in India has shown that only 48 and 43% of urban and rural patients (15-49 years) affected with hypertension are aware of their status, respectively [14]. Compared to the northern and western rural India, considerable variation in the prevalence of hypertension (20-59%) was seen among individuals from eastern rural India with higher prevalence seen from Assam (owing to the indigenous prevalence of excess salt, alcohol, and Khaini consumption among tea plantation workers of Assam); Andaman and Nicobar Islands also projected high prevalence as compared with other southern rural parts of India [15]. A longitudinal study observing the trends in the prevalence, awareness, treatment, and control of hypertension for the past 25 years inferred that there’s an increase in the prevalence of hypertension in the elderly urban population of India. Still, with an increase in awareness, i.e., 13% in 1995 to 56% in 2015, there is an increase in treatment from 9 to 36%. However, the study reported low hypertension control rates, as it managed to attain only 21% by 2015 from 2% to 1995 [5].

Existing literature ascertains several barriers to effective management of hypertension at both macro and micro levels. These include low consultation rates, insufficient availability, accessibility, and affordability of antihypertensive medications [16]. Another such barrier is a higher intake of dietary sodium among those exceeding the cut points of hypertension, which hinders the management and control of BP [17].

Despite the growing peril of hypertension, there is a scarcity of nationally-representative empirical evidence on the burden, management, and implications of hypertension among the elderly population in India. This information is essential to develop comprehensive and effective public-health strategies to prevent and effectively manage hypertension. Thus, to suffice the existing dearth on the issue, this study aims to (1) quantify the magnitude of measured, aware, treated, and controlled hypertension cases and identify the stage with maximum loss (2) identify the co-morbidities attributed to uncontrolled blood pressures among the hypertensive elderly population and (3) to recognize and suggest lifestyle modifications to reduce the likelihood of uncontrolled BP among the elderly population of India.

## Data and Methods

### Study design

The present study employed data from the first wave of the Longitudinal Ageing Study in India (LASI), 2017-18. LASI is a nationally representative study on ageing and health, conducted under the Ministry of Health and Family Welfare (MoHFW), Government of India. International Institute for Population Sciences (IIPS) collaborated with Harvard T. H. Chan School of Public Health and the University of Southern California to implement the survey [18]. LASI collected information on individuals aged 45 years and above and their spouses who reside in the same household, irrespective of age. LASI combined information from personal interviews with standardized physical examinations and laboratory tests.

**Fig. 1:**
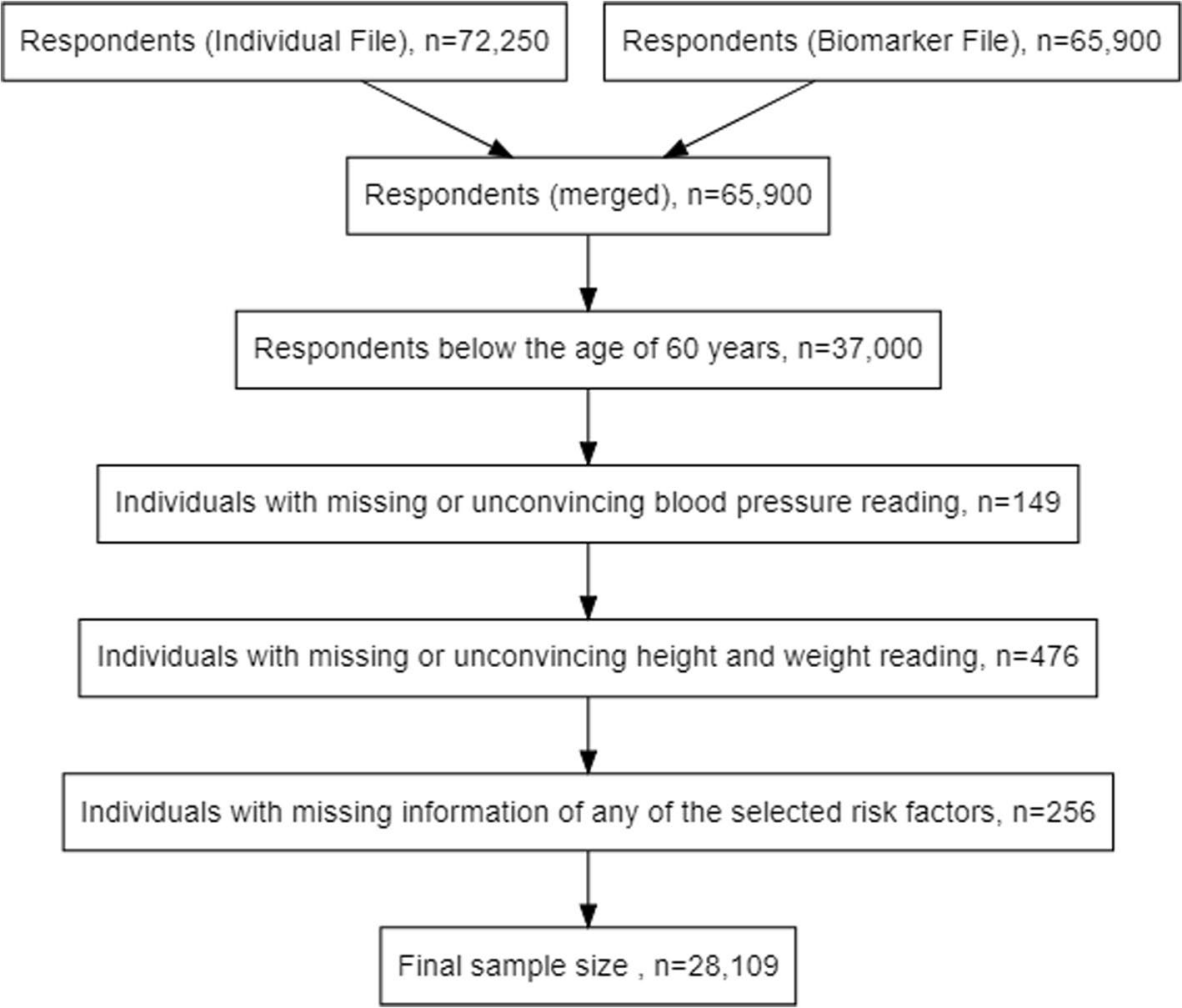
Selection of sample size (inclusion and exclusion criteria) from the Longitudinal Ageing Study in India, LASI, wave-1, 2017-18

The survey employed a multistage stratified area probability cluster sampling design and interviewed a total of 72,250 individuals [18]. The present analysis was restricted to elderly respondents, i.e., those aged 60 years and above. In addition, we employed a complete case analysis, and 28,019 respondents were included in the final analysis. A detailed description of the study sample is presented in Fig. 1.

### Measures

During the interview, self-reported hypertension, medication and family history of high blood pressure (BP) were asked. Three systolic and diastolic BP measurements were taken in a sitting position using an Omron HEM 7121 BP monitor during the physical examination, adopting internationally comparable protocols. Additional information on smoking, exercise and consumption of alcohol or food 30 min before to the blood pressure measurement were also collected.

An individual was classified as hypertensive by using the criteria formulated by the US Seventh Joint National Committee on Detection, Evaluation, and Treatment of Hypertension (JNC-VII), i.e., systolic BP (SBP) ≥ 140 mmHg and diastolic BP (DBP) ≥ 90 mmHg and those on anti-hypertensive medication [19]. BP levels were classified as usual if SBP<120 mmHg and DBP<80 mmHg and pre-hypertensive if 120 mmHg ≤SBP<140 mmHg or 80 mmHg ≤DBP<90 mmHg. These were categorized in three stages: stage-I (SBP 140–159 mmHg /DBP 90–99 mmHg), stage-II (SBP 160–179 mmHg /DBP 100–109 mmHg), and stage III (SBP ≥ 180 mmHg /DBP ≥ 110) mmHg [19] (see supplementary Figure S1).

We defined cascades in hypertension to see the discontinuations of patients at each stage i.e., whether they followed medical experts’ advice on not. We modify the framework to define this cascade in hypertension as defined by Wozniak et al. [20]. The cascades in hypertension care included measured, aware, treated, and controlled for elevated blood pressure.

Individuals who had SBP ≥ 140 mmHg or DBP ≥ 90 mmHg at the time of the survey were considered as ‘Measured cases of hypertension’. Whereas, individuals who responded affirmatively to the question, ‘Has any health professional ever told you that you have hypertension or high blood pressure?’, were considered as ‘Aware’ cases [18]. ‘Treated’ was defined among hypertensive population as currently taking medication to lower BP, based on affirmative responses to the questions: “To control your blood pressure or hypertension, are you currently taking any medication?”[21]. Finally, ‘control’ was defined as consuming anti-hypertensive medication and having an average SBP below 140 mmHg or DBP below 90 mmHg [18]. The outcome “uncontrolled” were defined as the complementary of “controlled cases”.

For identification of associates, the study incorporated selected background characteristics as independent variables. This included age (in years), sex, years of schooling, social group, religion, living arrangement, monthly per capita expenditure (MPCE) quintile, place of residence, self-reported health, family history of hypertension, and working status.

For identification of the co-morbidities attributed to uncontrolled blood pressures, information on seven NCDs, diabetes, lung cancer, heart disease, stroke, musculoskeletal disorders, neurological disorders, and high cholesterol were included. All these co-morbidities were self-reported in nature and were coded in binary form of present and absent.

For suggesting possible solutions, the study incorporated information on six modifiable risk factors available in the LASI dataset. These include current smoking status (no/yes), not following diet restrictions (no/yes), physically active (no/yes), regular alcohol consumption (no/yes), obesity (no/yes), and abdominal obesity (no/yes).

Central obesity (obesity) was defined as Body Mass Index (BMI) ≥ 30 kg/m^2^. BMI was calculated as weight (in kgs) divided by the square of height (in m^2^). Whereas, abdominal obesity was measured using Waist-Hip Ratio (WHR). WHR was calculated as waist circumference (in cm) divided by hip circumference (in cm). No (low risk) included WHR<0.90 for men and WHR<0.85 for women, while yes (high risk) included WHR≥ 0.90 for men and WHR ≥ 0.85 for women.

### Statistical analysis

Descriptive statistics were presented from the study. The summary statistics of the study sample by selected correlates were reported via two-way cross-tables. Multivariable logistic regression models were used to assess the association of hypertension and uncontrolled hypertension with selected background characteristics. Model coefficients were tested at a 5% level of significance. Relative risks (RR) based on post-estimations from these regression models were used to compute population attributable risk (PAR) for hypertension and uncontrolled hypertension.

In our study, PAR (expressed in percentage) depicts the proportion of hypertension and uncontrolled hypertension attributed to the selected predictor(s). For example, it demonstrates the extent to which hypertension and uncontrolled hypertension can be reduced in an ideal situation where the selected predictor(s) is set to its best condition. Several studies have estimated PAR to assess the impact of risk factors on hypertension in different scenarios [22, 23]. In our analysis, PAR estimates were based on all predictors included in the study.

The estimations were computed using Stata version 15.0 (College Station, Texas) [24]. The estimates provided in the present study were computed after applying suitable sampling weights provided by LASI wave-1, 2017-18 [18]. The study followed the Strengthening the Reporting of Observational Studies in Epidemiology (STROBE) reporting guidelines for cross-sectional studies (Supplementary Table 1).

## Results

### Background characteristics

Table 1 presents the descriptive statistics of the study sample. Approximately 31% of study individuals belonged to 60-64 years (30.69%). The study sample was dominated by the female (53% vs. 47%) population. About 56% of the respondents had no education, and 19% belonged to the Scheduled Castes. The sample was predominately rural (72%) and Hindu religion (83.8%). 42% of the respondent were living with their spouse and children.

**Table 1:** Summary statistics of the study sample by population subgroups, India, LASI-1, 2017-18

Background characteristics	Weighted Percentage	Frequency
**Age (in years)**		
60-64 yrs.	30.69	9221
65-69 yrs.	29.42	7996
70-74 yrs.	18.77	5106
74-79 yrs.	10.90	2966
80 & above	10.22	2730
**Sex**		
Female	52.57	14,525
Male	47.43	13,494
**Years of schooling**		
No schooling	56.46	14,967
Less than 5 yrs. complete	11.73	3447
5-9 yrs. complete	17.99	5419
10 or more yrs. complete	13.83	4186
**Social group**		
Scheduled caste (SC)	18.97	4574
Scheduled tribe (ST)	8.00	4624
Other Backward Class (OBC)	45.56	10,656
None of the above	27.47	8165
**Religion**		
Hindu	82.66	20,505
Muslim	10.89	3315
Christian	2.80	2821
Others	3.65	1378
**Living arrangements**		
Alone	5.71	1456
With spouse &/or others	19.87	5493
With spouse & children	41.78	12,238
With children & others	27.54	7409
With others only	5.10	1423
**MPCE quintile**		
Poorest	21.57	5713
Poorer	21.65	5791
Middle	20.86	5765
Richer	19.51	5521
Richest	16.42	5229
**Place of residence**		
Rural	71.53	18,669
Urban	28.47	9350
**Self-reported health**		
Good	31.15	9478
Moderate	45.36	12,226
Poor	23.50	6297
**Family history of hypertension**		
No	81.26	22,377
Yes	18.74	5642
**Working status**		
Currently working	31.89	8517
Worked in past but currently not working	41.87	11,754
Never worked	26.24	7748
**Total**	**100.00**	**28,019**

### Classification of blood pressure levels

**Fig. 2:**
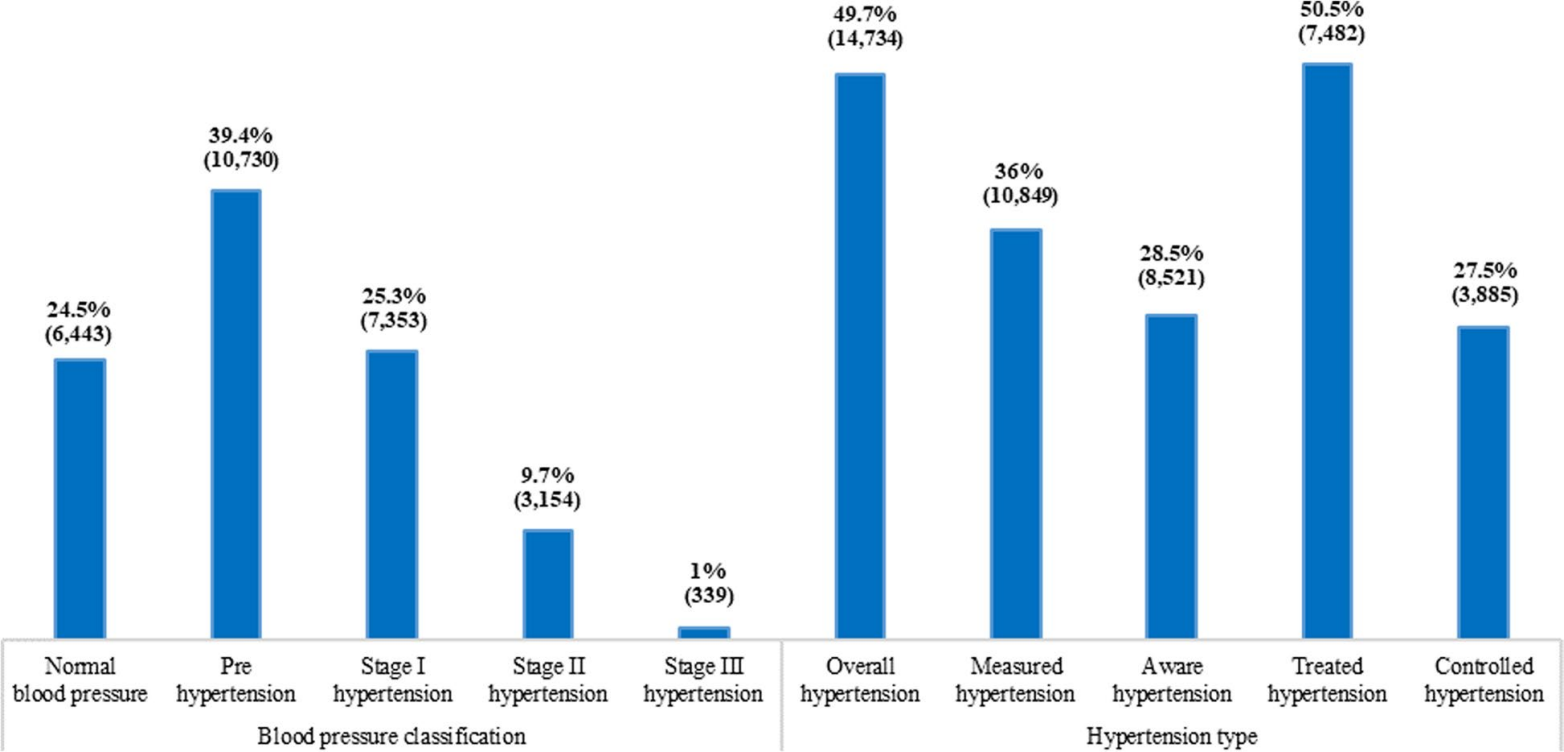
Classification of blood pressure level along with overall rates of prevalence, measured, aware, treatment and control of hypertension among elderly in India, LASI, waave-1, 2017-18

Figure 2 provides the classification of the BP levels. The prevalence of normal BP and pre-hypertension in the study sample was 25% and 39%, respectively. Approximately 25% of elderly people had stage I hypertension, 10% had stage II hypertension, and only 1% had stage III hypertension.

The proportion of measured, aware, treated, and control of hypertension is also shown in Fig. 2. 50% of the elderly had hypertension. About 36% of the elderly were measured for hypertension at the survey interview (measured for hypertension). Approximately 29% reported that they had raised BP (aware). Among all hypertensive individuals, 51% were currently on anti-hypertension medication. Out of all hypertensive individuals, 27.5% had their BP under control. Note that the measured and aware were the percent among all study participants, whereas treatment and control rates were explicitly calculated specifically for the hypertensive elderly population.

### The cascades of hypertension care

**Fig. 3:**
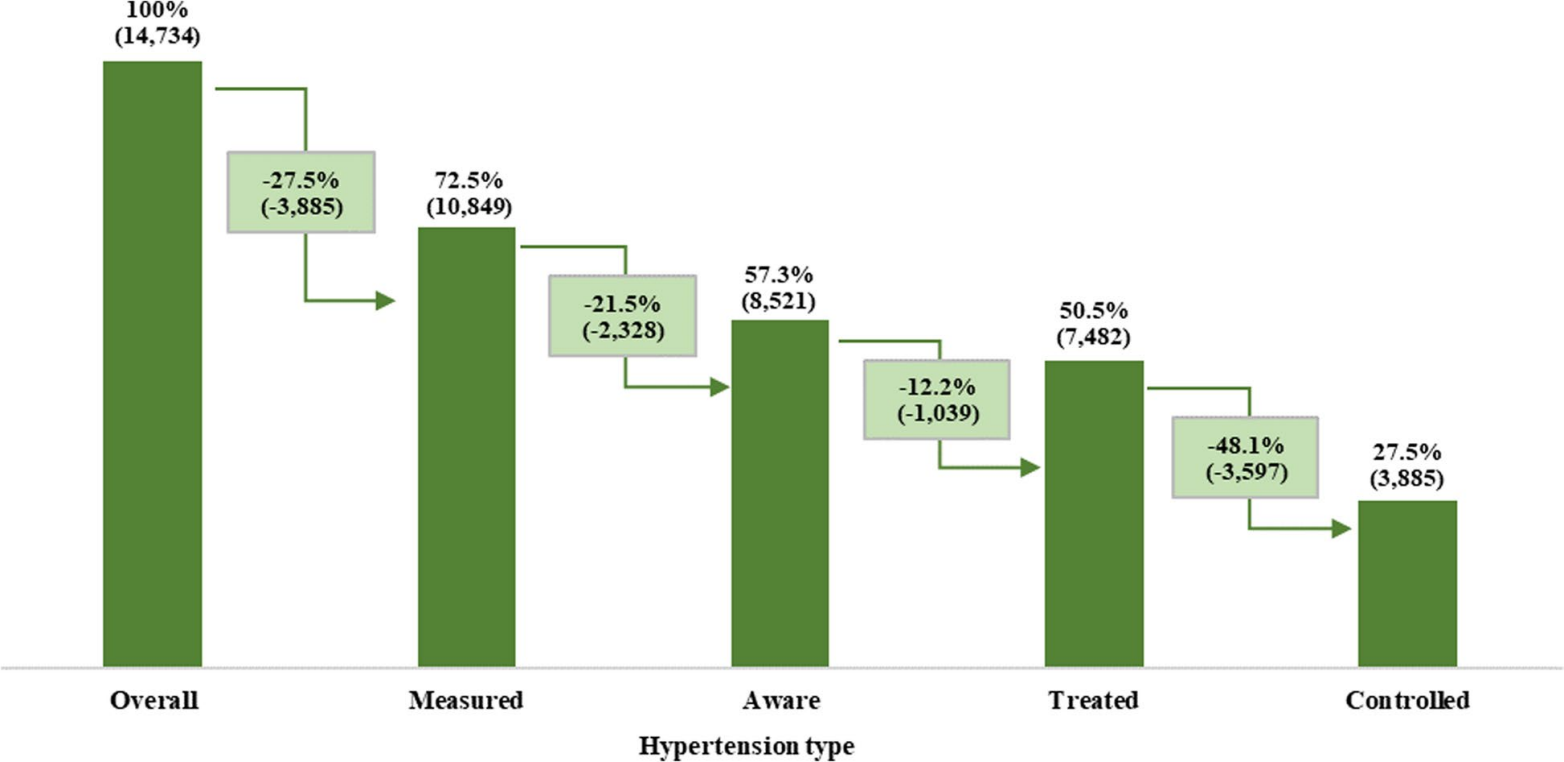
Cascade care of hypertension among elderly in India, LASI, wave-1, 2017-18

Figure 3 presents a flow chart that shows the percent of individuals receiving care at each stage of the hypertension cascade. Out of total hypertensive individuals, almost 73% were measured at the time of the survey for hypertension, a loss of 27% at the first stage. Among them, who had been measured, 78.5% were aware (self-reported) of hypertension. It confirms a loss of 21.5% at the second stage. Furthermore, among those aware of hypertension, 87.8% went for effective treatment. It describes a loss of 12.2% at the treatment stage. Among those who were under the medication for hypertension, 51.9% had their BP in control. Again, a loss of 48.1% at the control stage. These outcomes confirm a significant loss at each level of the hypertension care cascade. Therefore, the unmet need for hypertension care was estimated to be 72.5% among the hypertensive elderly in India.

### Hypertension and its associates

Table 2 depicts the logistic regression models for hypertension and uncontrolled hypertension. Model-I identifies the risk factors for hypertension. Results reveal that age, years of schooling, caste, religion, living arrangement, residence, family history of hypertension, working status, smoking, alcohol consumption, and obesity were significantly associated with hypertension. The odds for hypertension had shown an inclining trend with an increase in age. The odds of having hypertension were 24% (AOR=1.24; 95% CI: 1.11-1.37) more likely in the 80 years and above age group than in the 60-64 years age group. Individuals with ten or more years of schooling showed the odds of having hypertension 1.29 (AOR=1.29; 95% CI: 1.17-1.42) times more likely than individuals with no schooling. Muslim individuals have 17% (AOR=1.17; 95% CI: 1.07-1.28) higher odds of having hypertension than Hindu. Individuals who belong to the urban areas have 1.24 (AOR=1.24; 95% CI: 1.16-1.31) times higher odds of having hypertension than rural areas.

**Table 2:** Estimated odds ratio for hypertension and uncontrolled hypertension, LASI, wave-1, 2017-18

Background characteristics	Overall hypertension		Uncontrolled hypertension	
	**Model I**		**Model II**	
	**AOR**	**95% CI**	**AOR**	**95% CI**
**Age (60-64 yrs.) ®**	**1.00**		**1.00**	
65-69 yrs.	1.09*	[1.02 - 1.17]	0.98	[0.88 - 1.09]
70-74 yrs.	1.23***	[1.13 - 1.33]	0.95	[0.84 - 1.07]
74-79 yrs.	1.28***	[1.16 - 1.41]	1.14	[0.98 - 1.33]
80 yrs. & above	1.24***	[1.11 - 1.37]	1.11	[0.94 - 1.30]
**Sex (Female)®**	**1.00**		**1.00**	
Male	0.97	[0.90 - 1.05]	1.17*	[1.04 - 1.31]
**Years of schooling (No schooling) ®**	**1.00**		**1.00**	
Less than 5 yrs. complete	1.16***	[1.07 - 1.27]	0.94	[0.82 - 1.08]
5-9 yrs. complete	1.18***	[1.09 - 1.28]	1.03	[0.92 - 1.16]
10 or more yrs. complete	1.29***	[1.17 - 1.42]	0.96	[0.84 - 1.10]
**Social group (Scheduled caste (SC)) ®**	**1.00**		**1.00**	
Scheduled tribe (ST)	1.23***	[1.11 - 1.36]	1.70***	[1.42 - 2.04]
Other Backward Class (OBC)	0.93	[0.86 - 1.01]	0.89	[0.78 - 1.02]
None of the above	1.03	[0.94 - 1.12]	0.92	[0.80 - 1.06]
**Religion (Hindu)®**	**1.00**		**1.00**	
Muslim	1.17***	[1.07 - 1.28]	1.22**	[1.07 - 1.39]
Christian	1.17**	[1.05 - 1.30]	1.01	[0.86 - 1.18]
Others	1.27***	[1.11 - 1.44]	1.08	[0.89 - 1.29]
**Living arrangements (Alone)®**	**1.00**		**1.00**	
With spouse &/or others	0.71***	[0.62 - 0.81]	0.74**	[0.59 - 0.91]
With spouse & children	0.65***	[0.57 - 0.74]	0.73**	[0.59 - 0.89]
With children & others	0.85*	[0.75 - 0.97]	0.88	[0.72 - 1.08]
With others only	0.88	[0.74 - 1.04]	1.06	[0.82 - 1.38]
**MPCE quintile (Poorest)®**	**1.00**		**1.00**	
Poorer	0.94	[0.87 - 1.02]	0.93	[0.81 - 1.08]
Middle	1.02	[0.94 - 1.11]	0.89	[0.77 - 1.02]
Richer	1.01	[0.93 - 1.10]	0.76***	[0.66 - 0.88]
Richest	0.96	[0.88 - 1.05]	0.68***	[0.59 - 0.79]
**Place of residence (Rural)®**	**1.00**		**1.00**	
Urban	1.24***	[1.16 - 1.32]	0.72***	[0.66 - 0.79]
**Self-reported health (Good)®**	**1.00**		**1.00**	
Moderate	0.95	[0.89 - 1.05]	0.91	[0.83 - 1.01]
Poor	0.98	[0.90 - 1.05]	0.78	[0.70 - 0.88]
**Family history of hypertension (No)®**	**1.00**		**1.00**	
Yes	1.17***	[1.09 - 1.26]	0.90*	[0.82 - 0.99]
**Working status (Currently working) ®**	**1.00**		**1.00**	
Worked in past but currently not working	1.14***	[1.06 - 1.22]	0.88*	[0.78 - 0.99]
Never worked	1.10*	[1.00 - 1.19]	0.89	[0.78 - 1.03]
**Note**: *p<0.05, **p<0.01, ***p<0.001; AOR-Adjusted Odds Ratio; CI-Confidence interval; ®-Reference category.				

### Uncontrolled hypertension and its predictors

Model-II (Table 2) illustrates the risk factors for uncontrolled hypertension. Eight variables, caste, religion, living arrangement, MPCE quintile, residence, family history of hypertension, working status, and alcohol consumption, were significant predictors of uncontrolled hypertension in the multivariable logistic regression model.

Male hypertensive individuals had 1.17 times more risks of uncontrolled hypertension than female hypertensive individuals (AOR = 1.17, 95% CI: 1.04–1.31). Hypertensive patients living with their spouse and others had a 26% less risk of uncontrolled hypertension (AOR = 0.74, 95% CI: 0.60–0.91) compared to those living alone. Hypertensive individuals from Scheduled Tribes had 1.70 times more risk of uncontrolled hypertension than hypertensive individuals from Scheduled Castes (AOR = 1.70, 95% CI: 1.42–2.04). Hypertensive individuals from the Muslim religion had 1.22 times more risk of uncontrolled hypertension than hypertensive individuals from the Hindu religion (AOR = 1.22, 95% CI: 1.07–1.39). Hypertensive patients who belonged to the richest quintile had 33.80% less risk of uncontrolled hypertension (AOR = 0.68, 95% CI: 0.59–0.79) than those who belong to the poorest quintile. Hypertensive respondents from the urban area had a 27.7% less risk of uncontrolled hypertension (AOR = 0.72, 95% CI: 0.66–0.79) than those from the rural areas. Similarly, hypertensive individuals who had a family history of hypertension had a 9.6% less risk of uncontrolled hypertension (AOR = 0.90, 95% CI: 0.82–0.99) compared to those who didn’t have a family history of hypertension.

### Health Deteriorating Implications: Linkages between hypertension and selected NCDs

Association of hypertension with seven recurrent chronic comorbidities are shown in Table 3. It is worth mentioning that all the diseases included in the study, diabetes, lung cancer, heart disease, stroke, musculoskeletal disorders, neurological disorders, ang high cholesterol were self-reported and were coded into binary form. Here absence of disease was considered as the reference category. Among the hypertensive individuals, the chance of having diabetes (75%) at the same time is highest as compared to other chronic conditions, then followed by heart diseases (73%) and strokes (70%). Across all the concerned chronic morbidities, the PAR value was the highest for cholesterol, with a PAR value of 7.47% (95% CI: 3.40; 11.53) for hypertension. (i.e., if hypertension is eliminated from the population, then a chance of developing high cholesterol is minimized by 7.47%). If hypertension is eliminated from the population, then the risk of being diabetic would be reduced by 6.42% (95% CI: 3.98; 8.85). Similarly, lung cancer and heart diseases would be reduced by 5.10% and 5.03%, respectively.

**Table 3:** Estimated odds ratio and population attributable risk (PAR) for individuals with chronic non-communicable diseases and having hypertension, LASI, wave-1, 2017-18

Chronic morbidities	Prevalence (per 100 population)	AOR	95% CI	PAR%	95% CI
Diabetes	74.89	1.47***	[1.35, 1.61]	6.42	[3.98, 8.85]
Lung cancer	51.97	0.88***	[0.79, 0.98]	5.10	[2.92, 7.28]
Heart disease	73.06	1.19***	[1.02, 1.37]	5.03	[2.82, 7.23]
Stroke	69.78	1.65***	[1.34, 2.04]	2.79	[2.18, 3.39]
Musculoskeletal Disorder	55.89	0.96***	[0.89, 1.03]	3.45	[2.50, 9.41]
Neurological Disorders	55.50	1.06***	[0.88, 1.27]	0.76	[1.68, 3.19]
High Cholesterol	68.77	1.39***	[1.15, 1.68]	7.47	[3.40, 11.53]

Note

1) *p<0.05, **p<0.01, ***p<0.001

2) AOR-Adjusted Odds Ratio; CI-Confidence interval; PAR- Population Attributable Risk

3) All the AOR and PAR values are adjusted for all the risk factors and other socio-demographic variables

4) In case of AOR reference category is the absence of hypertension

### Modifiable predictors and their contribution to the burden of hypertension and uncontrolled hypertension

Table 4 presents PAR estimates for hypertension and uncontrolled blood pressure on all risk factors. We have included all possible risk factors of hypertension and uncontrolled blood pressure levels, namely currently smoking, diet restrictions, physically active, regular alcohol consumption, obesity, and abdominal obesity. The reference category for each of the discussed risk factor was their counterfactual, for instance in case of currently smoking, those who are not currently smoking are reference category. The overall prevalence of uncontrolled hypertension in the study was 72.50%. If all the concerned risk factors are eliminated from the population, in the ideal situation (counterfactual), a possible reduction by 14.60% points (95% CI: 13.66; 15.67) in hypertension and 23.89% points (95% CI: 22.30; 25.01) in uncontrolled hypertension can be achieved. Across all the risk factors, the PAR value was the highest for diet restriction, with a PAR value of 12.19% (95% CI: 11.92; 12.46) for hypertension and 18.64% (95% CI: 17.79; 19.48) for uncontrolled hypertension. If the obesity was eliminated, then the risk of being hypertensive would be reduced by 2.16% point (95% CI: 1.78; 2.55). Similarly, if alcohol consumption was eliminated from hypertensive individuals, the risk of uncontrolled hypertension is reduced by a 4.31% point (95% CI: 0.73; 7.89).

**Table 4:** Estimated adjusted odds ratio and population attributable risk (PAR) for hypertension and uncontrolled hypertension for modifiable risk factors, LASI, wave-1, 2017-18

Risk factors	Hypertension			Uncontrolled Hypertension		
	**Prevalence**	**AOR** **(95% C.I.)**	**PAR%** **(95% C.I.)**	**Prevalence**	**AOR** **(95% C.I.)**	**PAR %** **(95% C.I.)**
Currently smoking	41.39	0.79*** (0.73, 0.85)	-0.65 (-0.88, -0.42)	79.71	0.99 (0.86, 1.16)	0.02 (-2.05, 2.09)
Not following diet restriction	92.78	1.03** (0.97, 1.09)	12.19 (11.92, 12.46)	50.14	1.53** (1.04, 1.76)	18.64 (17.79, 19.48)
Physically active	47.12	1.03 (0.96, 1.11)	0.13 (-0.14, 0.39)	77.29	0.99 (0.87, 1.12)	0.18 (-1.44, 1.79)
Regular alcohol consumption	49.68	1.35*** (1.12, 1.54)	0.24 (0.13, 0.35)	83.82	1.352* (1.04, 1.76)	4.31 (073, 7.89)
Obese(BMI)	73.71	1.77*** (1.60, 1.96)	0.53 (0.36, 0.71)	65.14	1.06 (0.95, 1.19)	0.56 (0.96, 2.09)
Have abdominal Obesity (waist/height)	66.04	1.13 (0.89, 1.22)	2.16 (1.78, 2.55)	66.57	1.18** (0.95, 1.42)	0.18 (0.73, 1.09)
**Total**	**49.70**	**-**	**14.60** **(13.66, 15.67)**	**72.50**	**-**	**23.89** **(22.20, 25.01)**

## Discussion

### Summary of the findings and possible mechanism

This study rests on a nationally representative sample of 28,019 elderly in India. The findings depict an enormous hypertension burden, with every second elderly individual affected in India. Hypertension has been reported as one of the most prevalent chronic non-communicable diseases among older adults in India [25, 26], with a wide variety of detrimental implications ranging from social, economic, and psychological. In addition, existing literature establishes hypertension as a primary disease in forming morbidity clusters predominantly among older adults at the national level [25, 27].

A significant proportion of the population was lost as we transit from one stage of hypertension care cascades to another; however, the highest relative loss was observed at the level of control (48.1%). Therefore, the study findings ascertain that the existing care cascades are inadequate to effectively prevent and manage the emerging hypertension burden, with the particular focus required at the control stage. Effective management of hypertension can serve as a primary precursor to improved health-related outcomes, reduced risk of organ damage [28], and development of comorbidities and polypharmacy among the elderly population in India [29]. Few studies have earlier highlighted the insufficiency of hypertension care cascades, but they were either based on small samples [30–32] or included different age brackets [14]. In the studies providing national estimates, awareness and treatment stages reported the highest loss, primarily focusing on the working population [12, 14]. Whereas the elderly presents a distinctive scenario with extremely high hypertension burden and mediocre care cascades, with the highest loss reported at the level of control.

The study findings establish that socio-economic, demographic, and behavioral risk factors influence the burden of both hypertension and uncontrolled BP levels. The findings suggest that with increasing age, higher levels of schooling, the burden of hypertension increases. Additionally, the burden was higher for the individuals who are obese or are binge-alcoholics from the urban areas. Existing medical literature presents hypertension as a multifactorial disease, which means that the disease lack monocausal etiology and can be considered an amalgamation of multiple interacting causes, including socio-economic and demographic, genetic, behavioral, vascular, and neuroendocrine risks [33]. As hypertension is primarily a lifestyle disease, the elderly leading an urbanized lifestyle which majorly comprises a high-energy diet rich in carbohydrates, sugar, and trans-fats; and prominently low level of physical activity, possess a considerably higher burden of hypertension [15, 34]. This urbanized lifestyle often leads to overweight and obesity, which is further catalyzed by a large amount of alcohol consumption [31, 34, 35].

The findings also suggest that elderly men and those living alone have a higher risk of having uncontrolled BP. These findings are in concordance with the studies done in the past, which suggest that hypertension control is lower among men than women, primary reasons being low consultation rates and poor adherence to diet and medications, and unhealthy behaviors like smoking, drinking, and leading a sedentary lifestyle which is higher among men than women in India [12, 14, 36, 37]. Existing literature has reported that loneliness can cause and exacerbate the risk of cardiovascular diseases, including high systolic blood pressure and cholesterol among various sub-groups; however, these results are intensified in the case of the elderly, who are more prone to experience loneliness than younger individuals [38, 39]. In addition to this, those belonging to the rural areas, and the poorest wealth quintile has higher risk of getting affected with uncontrolled blood pressures. This could be due to the lower awareness, availability, accessibility and affordability of aggressive medical treatment [14].

Family history was testified to play a vital role in lowering the risk of uncontrolled BP; this could be attributed to the fact that hypertension is usually asymptomatic. This makes accepting the diagnoses extremely difficult. However, having a family history of the disease increases the acceptance of certain diagnoses and makes one naturally more informed about managing the same effectively. Obesity, on the other hand; has been reported to increase the risk of uncontrolled BP manifolds. Medical literature has proposed several pathways in which high body fat or obesity can elevate the blood pressure levels in humans, these include damaged renal-pressure natriuresis because of physical compression of the kidneys and activation of the RASS and SNS [40–42]. Thus, treatment guidelines need to be positioned to reduce obesity-associated BP levels, and advocacy of weight reduction by the physicians.

Existing literature presents hypertension as a bridge between several disease clusters in India [27]. The findings from our study hint that if hypertension is controlled adequately, one can reduce the risk of high cholesterol, diabetes, lung cancer, and heart disease, which can reduce the burden on the country’s fragile healthcare infrastructure. While heart disease and high cholesterol lie under the common umbrella of cardiovascular diseases [43], hypertension is reported to pose a serious threat to individuals who have diabetes and vice-versa. Uncontrolled blood pressure can severely damage the inner walls of arteries, which in turn decreases the stream of blood and oxygen to the heart, which often leads to heart disease [43]. On the other hand, high cholesterol levels are reported to narrow and harden the blood vessels, which increases the pressure on the heart and, in turn, causes elevated blood pressures [44, 45]. Diabetes is portrayed to have a bi-directional relationship with hypertension [44]; additionally, diabetes among hypertensive individuals is reported to catalyze cardiovascular diseases, like heart diseases and stroke [44].

### Strengths and Limitations

The study attempts to present empirical evidence on hypertension care cascades among the Indian elderly, which by far has been overlooked. The study’s major strength is using nationally representative data, LASI, which has sufficed the existing shortage of large-scale data on the elderly population in the country.

Despite the aforementioned strengths, the actual loss of patient at each stage of hypertension care cascade is not measured and the study did not capture temporal associations as the study was based on cross-sectional data, which might otherwise be utilitarian. As the study focuses on the elderly population, all the estimates presented in the study should be generalized specifically for the same sub-group of the country’s population.

### Implications of the findings

The findings from the study can be used in two ways; firstly, the information from the cascades can be used to design hypertension management interventions for the elderly population in the country. Secondly, the findings highlight the patients’ sub-group with inefficient hypertension control, i.e., ineffective treatment, including men, scheduled tribes, Muslims, living alone, rural areas, family history of chronic disease, and currently working elderly. The primary reasons for ineffective treatment are patient-centered factors and medical causes of secondary hypertension that can be explored by implementing streamlined patient referral pathways in the newly established Health and Wellness Centres (HWCs) [20]. Physicians need to design effective control strategies, including increasing personal competence by cultivating communications between patient and healthcare provider, medication reminders, and assessing therapeutic and dietary adherence by the spouse, family members, or other relatives. As a solution to the identified burden of uncontrolled blood pressures, our findings suggest some possible suggestions based on the study’s empirical evidence. The study highlights adherence to dietary restrictions, eradicating obesity, and alcohol consumption to reduce the emergent peril of uncontrolled hypertension which is widely accepted worldwide [14, 44, 46].

## Conclusions

In conclusion, the elderly population in India experiences loss at all the four stages of hypertension care, with the highest dip reported at the level of blood pressure control. Thus, there is an urgent need to improve access to cost-effective anti-hypertensive prescriptions among the socially and economically disadvantaged sub-groups to curtail the increasing burden of uncontrolled BP. Furthermore, the study identifies the factors associated with hypertension and uncontrolled BP, which can assist in identifying the high-risk sub-groups and devise more elderly-oriented interventions. To summarize, our study gives a holistic description of hypertension care cascades, and their linkages with prominently occurring comorbidities. Furthermore, the study identifies behavioral risk factors that can be modified to improve the burden of uncontrolled hypertension, thus, acting as a possible solution. If apprehended cautiously, findings from this study can serve to design effective approaches aimed at controlling, preventing, and managing hypertension among the elderly population in India.

## Supplementary Information

**Additional file 1.**

## Data Availability

The dataset analysed during the current study are available in the Longitudinal Ageing Study in India (LASI) repository, https://iipsindia.ac.in/sites/default/files/LASI_DataRequestForm_0.pdf. held at the International Institute for Population Sciences (IIPS), Mumbai. The access to the data requires registration which is granted specifically for legitimate research purposes.

## Abbreviations

AOR: Adjusted Odds Ratio
BP: Blood Pressure
CI: Confidence Interval
LASI: Longitudinal Ageing Study in India
NCD: Non-Communicable Diseases
PAR: Population Attributable Risk
